# Supramolecular assembly of unstructured peptides into rigid bundlemer polymers

**DOI:** 10.1039/d5nr03269e

**Published:** 2025-10-23

**Authors:** Joshua E. Meisenhelter, Matthew Langenstein, Jacquelyn E. Blum, Dai-Bei Yang, Darrin J. Pochan, Jeffery G. Saven, Christopher J. Kloxin

**Affiliations:** a Department of Chemical and Biomolecular Engineering, University of Delaware 150 Academy Street Newark DE 19716 USA cjk@udel.edu; b Department of Materials Science and Engineering, University of Delaware 201 DuPont Hall Newark DE 19716 USA; c Department of Chemistry, University of Pennsylvania 231 S. 34th Street Philadelphia PA 19104 USA saven@sas.upenn.edu

## Abstract

Unstructured, designed 15-residue peptide sequences conjugated between their N-termini through thiol–maleimide click chemistry yield coiled-coil, rod-like polymers with widths of 2 nm and lengths exceeding 5 μm. The assembly process enables supramolecular polymer formation and is distinct from previously reported step-growth polymerization of well-structured coiled coils.

## Abstract 2

Designed 15-residue peptides that are unstructured in solution fold into coiled-coil, rod-like polymers 2 nm wide and over 5 μm long upon N-to-N conjugation through thiol–maleimide click chemistry.

## Graphical abstract

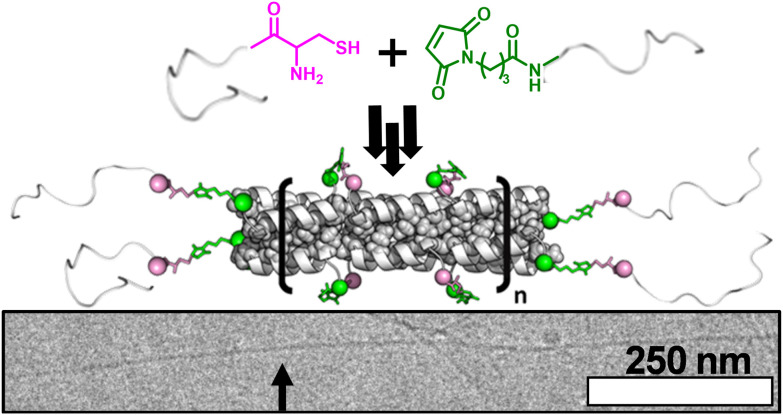

## Introduction

The supramolecular assembly of α-helix-forming peptides into oligomeric coiled coils can impart structural stability and dynamic functionality to both natural proteins^1,2^ and synthetic biomaterials, such as responsive hydrogels,^3,4^ molecular switches,^6^ and origami nanostructures.^7^ Coiled-coil peptides feature a repeating pattern of hydrophobic and hydrophilic residue interactions that spans seven amino acids, known as a heptad, in which two positions (*i*, *i* + 3) are typically occupied by hydrophobic amino acids.^8^ Under aqueous conditions, this arrangement drives α-helical folding with hydrophobic side chains oriented along one face of the helix, promoting amphiphilic assembly into a coiled coil. Structural stability increases with peptide length, and typically more than two heptads are required to form stable coiled coils^9,10^ in the absence of stabilizing interactions such as salt bridges, covalent staples, or other crosslinks.^11–13^ Probing the trade-offs between assembly efficiency and peptide length provides valuable insights into designing these biomaterials more effectively.

**Fig. 1:**
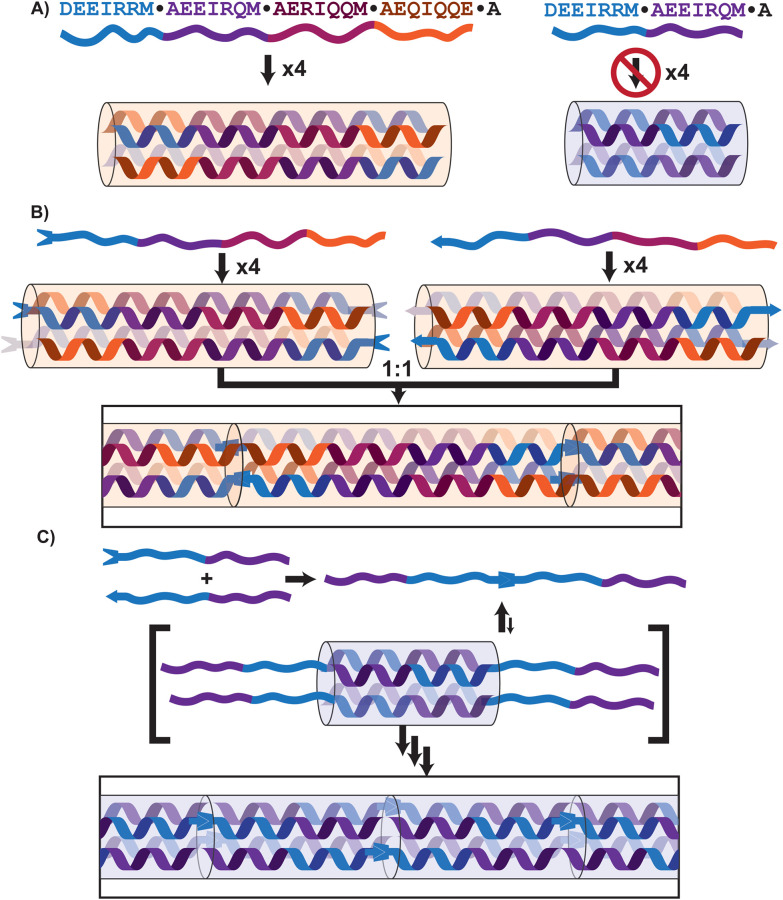
Illustration of bundlemer peptide assembly. The sequences are shown in single-letter amino acid code, with colours indicating individual heptads. (A) BNDL29, containing four putative heptads, assembles into a homotetramer coiled coil, whereas the truncated 15-residue BNDL15-TR, with only two heptads, does not form a stable structure. (B) In prior work, introduction of complementary click functional groups at the N-termini of stable bundlemers enabled their polymerization into rigid rods.^5^ (C) In this work, we hypothesize that the covalent linkage of two BNDL15-TR sequences will result in the transient formation of shorter coiled coils with unstructured, sticky ends can nucleate assembly into extended rod-like polymers.

Computational design has yielded synthetic coiled coils with exceptional stability over a range of temperature, pH, and salt conditions, enabling their use as building blocks for new materials. Designed coiled-coil bundles, or bundlemers, serve as supramolecular monomer units (Fig. 1). Computational methods facilitate *de novo* design and engineering, including control of external charges and hydrophobic interactions, to drive controlled assembly into intricate crystalline structures.^14–16^

Beyond physical (noncovalent) interactions, bundlemers can be chemically modified with thiol and maleimide ‘click’ functional groups to enable covalent linkage. In a previous design, antiparallel tetrameric bundlemers are functionalized at their N-termini such that each end of a bundlemer displays two click groups. When paired with another bundlemer bearing complementary click functionalities, the opposing ends react in a step-growth fashion, yielding covalent, ultra-rigid, rod-like polymers, illustrated in Fig. 1B.^5^ Structural models of these polymers reveal end-to-end alignment of neighbouring bundlemers, with alignment of α-helices and hydrophobic coiled-coil-core residues.^17^

We also showed that rods can be constructed from two different bundlemers with distinct thermal stabilities. In prior work, we combined one ultrastable bundlemer that remained folded above 90 °C with another that unfolded at a lower temperature.^5^ Heating to 90 °C disrupted the less stable component and disassembled the rods, while subsequent cooling enabled repolymerization. This reversible process demonstrated that rod assembly suggests a general principle of assembly *via* unstructured, “sticky” ends. We now test this principle directly using a truncated two-heptad system.

Herein, a previously characterized four-heptad (29-residue) coiled coil-forming peptide, BNDL29,^4,5^ was truncated to create a shorter, two-heptad (15 residues) variant: BNDL15-TR (Fig. 1A). BNDL29 is a computationally designed tetrameric coiled coil with an unfolding temperature exceeding 90 °C (see SI). Although peptides shorter than three heptads usually fail to form stable coiled coils,^4^ we hypothesized that N-to-N terminal conjugation of BNDL15-TR could enable polymeric formation by generating transient tetrameric intermediates with flexible, unstructured ‘sticky’ ends that promote further assembly into polybundlemers (Fig. 1C). Shortening the peptide length not only probes fundamental aspects of rod assembly but also offers practical advantages by improving synthetic yield and reducing costs.

## Results and discussion

### Peptide synthesis and characterization

**Fig. 2:**
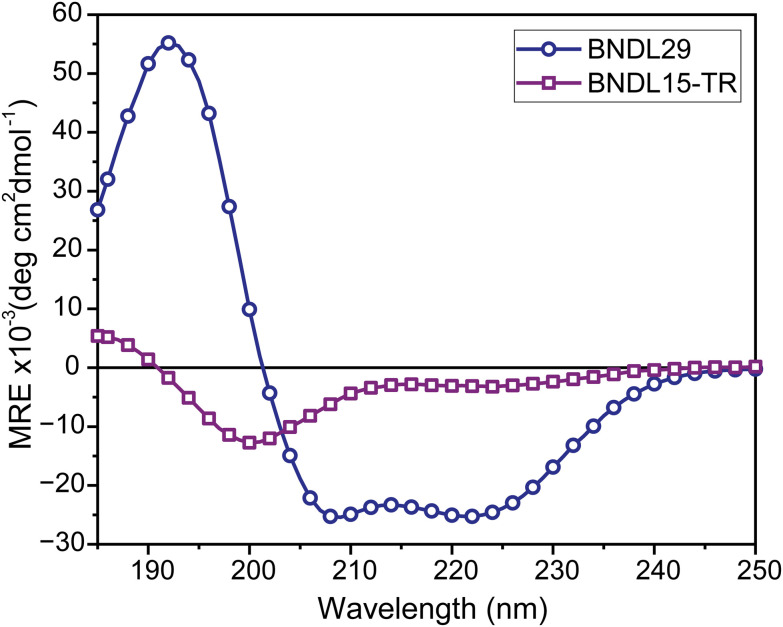
Circular dichroism spectra of BNDL29 and BNDL15-TR at 0.1 mM peptide concentration and 20 °C. The mean residue ellipticity (MRE) of BNDL29 (circles) revealed characteristic minima at 208 nm and 222 nm and a maximum at 195 nm, consistent with a helical structure. The ∼1 : 1 ratio of MRE at 208 nm and at 222 nm further supports coiled coils formation. In contrast, BNDL15-TR (squares) primarily displayed an MRE minimum near 200 nm, indicative of a random coil, with an additional shallow minimum at 222 nm and a maximum around 185 nm, suggesting limited helical structure.

BNDL15-TR, comprising residues 1–15 of BNDL29, was synthesized using standard solid-phase peptide synthesis (see SI for details). Circular dichroism (CD) spectra confirmed that BNDL29 exhibited characteristic minima at 208 and 222 nm (Fig. 2), consistent with a well-folded α-helical coiled coil. In contrast, BNDL15-TR lacked defined helicity, with a dominant minimum near 200 nm and <1% helix content by CD analysis.^18^ These results are consistent with the expectation that two-heptad sequences are unstructured unless stabilized by additional interactions.

### Rod polymerization

N-terminal chemical modifications were made to the BNDL29 and BNDL15-TR sequences by incorporating either cysteine (C) or 4-maleimidobutyric acid (Mal) during the final step of SPPS, yielding thiol- or maleimide-functionalized peptides (see SI details). Interestingly, these modifications increased the helicity of BNDL15-TR-C and BNDL15-TR-Mal to 16% and 22%, respectively (see Fig. S3 and Table S2). The increased helicity in BNDL15-TR-C may result from extension of the amide backbone, while BNDL15-TR-Mal may benefit from neutralization of the N-terminal positive charge. Despite these increases in helicity, both peptides remain predominantly unstructured.

**Fig. 3:**
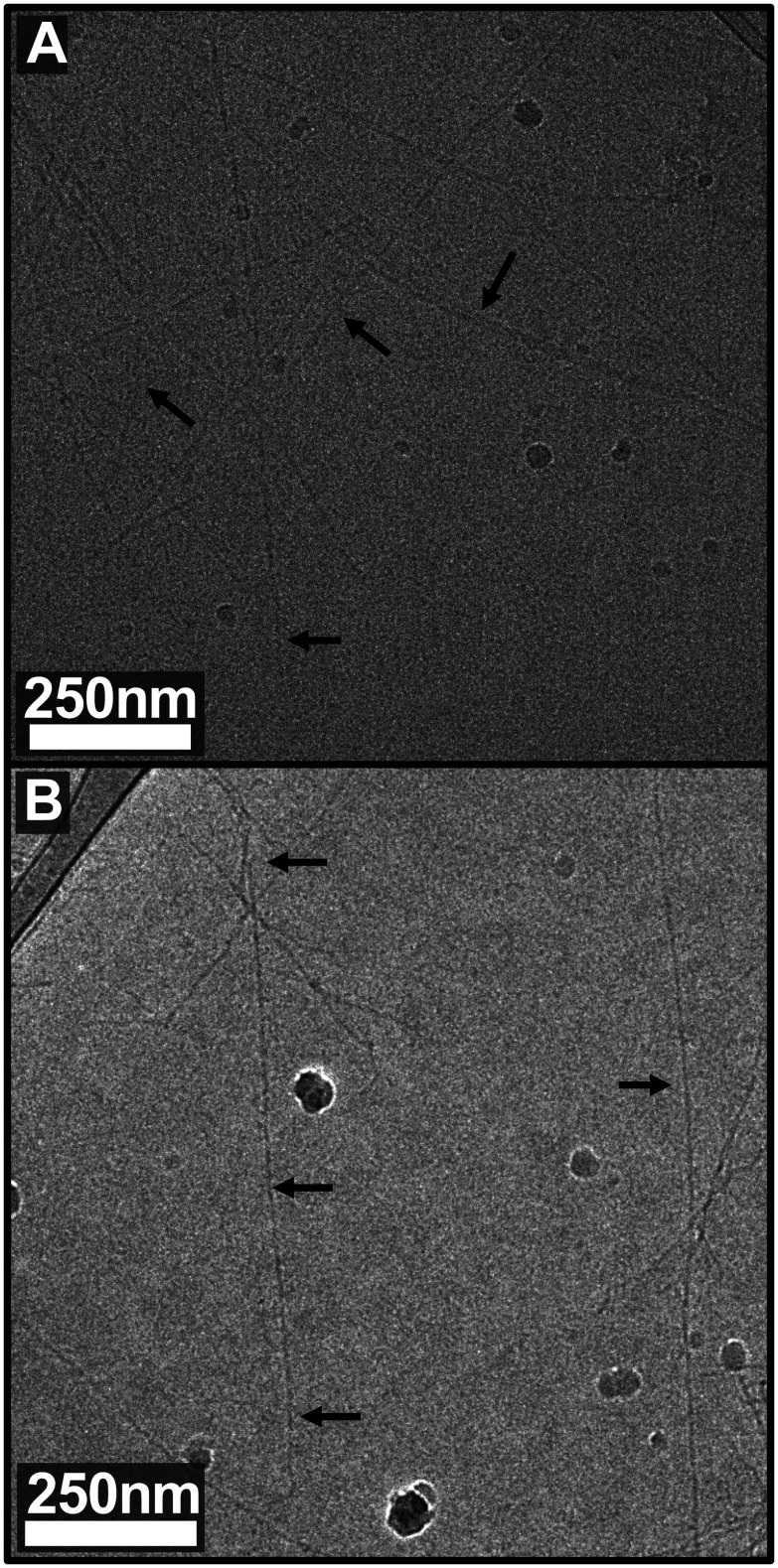
Cryo-TEM of rigid-rod polymers assembled from (A) BNDL29 and (B) BNDL15-TR. Peptides were synthesized with either a thiol or maleimide at the N-terminus and mixed in equimolar ratios to form rigid rod-like polymers. (A) Modified-BNDL29 formed rigid rods ∼2 nm in diameter and 1–2 μm in length. (B) Modified-BNDL15-TR formed rigid rods of comparable diameter (2 nm) but with lengths extending field of view of the microscope. For specimen preparation details and additional images at higher and lower magnification, see SI.

Consistent with previous work,^5^ equimolar mixtures of thiol- and maleimide-functionalized BNDL29 undergo step-growth polymerization *via* an A–A/B–B mechanism, forming rigid rods approximately 2 nm in diameter (*i.e.*, the width of the bundlemer), and extending to lengths of 1–2 μm (Fig. 3A). This process links peptides through N-to-N terminal conjugation at each bundlemer–bundlemer interface, in contrast to canonical N-to-C terminal ligation.^19^ The resulting polymers exhibit high rigidity and persistence lengths exceeding 10 μm,^5^ consistent with rods of colinear bundlemers.^17,20,21^ In this earlier system, polymerization was carried out at room-temperature, as BNDL29 peptides were already pre-assembled into stable coiled-coil bundles.

In contrast, BNDL15-TR peptides do not pre-assemble. We hypothesized that, although too short to form a stable tetrameric coiled coil in isolation, BNDL15-TR might still yield robust rod-like polymers if truncated sequences were covalently linked at their N-termini (Fig. 1C). To test this, BNDL15-TR-C and BNDL15-TR-Mal were reacted at 80 °C to drive rapid conjugation and minimize incorporation of unreacted peptides, then cooled to room temperature to initiate folding and rod extension. Cryogenic transmission electron microscopy (cryo-TEM) revealed rod-like structures approximately 2 nm in diameter, with some structures extending beyond 5 μm in length (Fig. 3B). The identical diameter to BNDL29-derived rods implies comparable coiled-coil packing and alignment, even though the isolated two-heptad BNDL15-TR peptide exhibits little intrinsic helicity.^4^

**Fig. 4:**
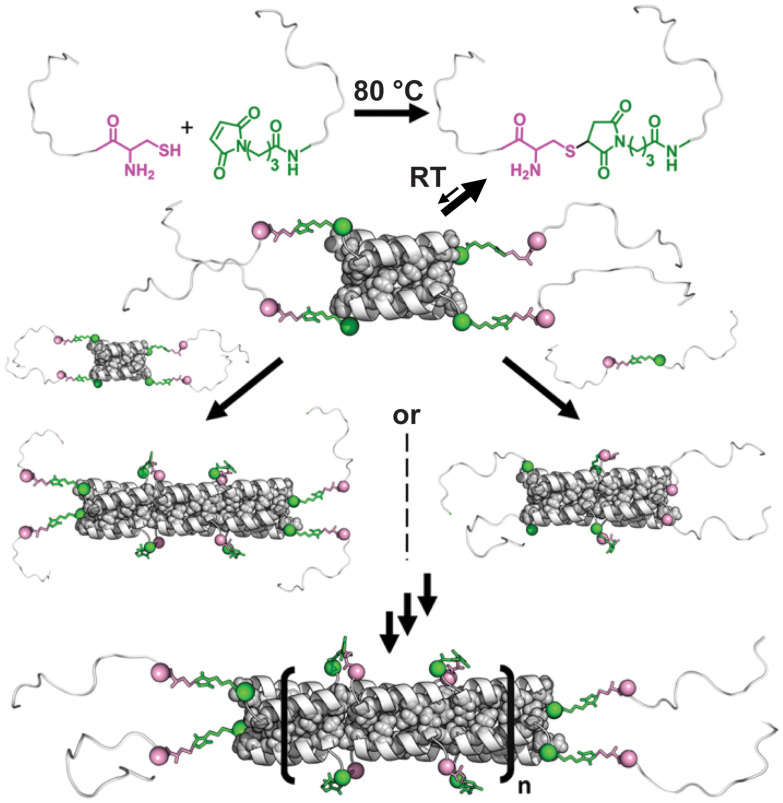
Scheme of BNDL15-TR rigid rod assembly. BNDL15-TR peptides were modified with either a thiol or maleimide group and conjugated at 80 °C. Upon cooling to room temperature, the resulting 30-residue conjugates transiently form coiled-coil bundlemers with two overhanging unstructured 15 residue segments at each end (‘sticky ends’). These flexible segments can associate with other transient bundlemers or free conjugated peptides to form additional, adjacent bundlemers, driving polymerization and rod-extension. This mechanism, polymerization *via* helical folding and association, produces longer rods than those observed by chemical conjugation of BNDL29. Interior hydrophobic residues (Ile and Ala, shown in space-filling representation) form a contiguous hydrophobic core across bundlemer interfaces.

The ability of the BNDL15-TR conjugate to assemble suggests a distinct rod formation mechanism compared to BNDL29. Whereas BNDL29 forms well-structured helical homotetramers that polymerize through stoichiometrically limited A–A/B–B step-growth reactions, rod length in this system is inherently capped by the ratio of reactive end groups, and even slight stoichiometric imbalances can significantly reduce the average length. In contrast, the covalently linked BNDL15-TR-C and BNDL15-TR-Mal peptides can form transient tetramers that present unstructured ‘sticky ends’ at both termini. These exposed segments enable continued recruitment of additional peptides or oligomers, driving rod elongation through supramolecular folding and axial extension (Fig. 4).

The resulting BLDL15-TR rods routinely exceed lengths observed for BNDL29, suggesting growth driven primarily by supramolecular folding and axial extension. While hierarchical synthetic polymer fibres can also achieve multi-micron persistence lengths, this generally involves lateral interactions or chain entanglement across multiple nanofibrils and growing fibres, leading to thicker and more heterogeneous diameters.^22,23^ In contrast, the uniform ∼2 nm diameter of BNDL15-TR rods suggests exclusively end-to-end assembly (Fig. 3).

Although BNDL15-TR is largely unstructured in isolation, its N-to-N conjugate forms rods exceeding 5 μm. Each rod terminus displays two 15-residue segments that recruit additional peptides, enabling further bundlemer formation and extension (Fig. 4). Here, chain elongation proceeds not by ‘click’ chemical reaction but by sequential folding of new tetrahelical units, bypassing limitations inherent to purely covalent step-growth polymerization and yielding extremely long bundlemer-width nanorods.

Models of N-to-N linked 29-residue peptides show colinear alignment of tetramer superhelical axes, continuous hydrophobic core packing across bundlemer interfaces, and alignment of N- and C-termini to maintain α-helical hydrogen bonding.^17^ This pseudo-contiguous superhelix suggests that similar rod-like assemblies can be achieved with short peptides, due to stabilizing interactions that arise during polymer growth (Fig. 4). Notably, the two-heptad BNDL15-TR, ordinarily a poor helix-former, is transformed into a robust rod-former upon N-to-N dimerization. BNDL15-TR lacks modification of the C-terminal residues that result from truncation, residues that are interior to the sequence of BNDL29 (Fig. 1). These findings highlight the remarkable tolerance of bundlemer assembly to sequence truncation and underscore the heptad modularity of this design. This strategy circumvents the three-heptad threshold typically required for stable coiled-coil formation and offers a shorter, synthetically advantageous 15-residue motif for constructing robust supramolecular nanomaterials.

## Conclusions

Excising the first 15 residues of the designed four-heptad bundlemer, BNDL29, yields a two-heptad variant, BNDL15-TR, which is unstructured (CD-based helicity ≈ 1%). Installing thiol or maleimide at the N-terminus partially restored some helicity (16–22%). When full-length BNDL29 was cross-linked through N-to-N thiol–maleimide chemistry, polymerization reproduced previous observations, yielding 2 nm-wide rods 1–2 μm in length. Strikingly, performing the same coupling with the BNDL15-TR thiol (BNDL15-TR-C) and maleimide (BNDL15-TR-Mal) analogues produced rods of identical diameter but routinely exceeded 5 μm, revealing a fundamentally different, assembly-driven growth mechanism rather than the stoichiometry-limited step-growth process observed with BNDL29.

Collectively, these results demonstrate that covalent N-to-N concatenation can bypass the long-standing three-heptad stability threshold in the context of rod formation, converting a largely unstructured two-heptad sequence into a durable coiled-coil building block. The uncommon N-to-N peptide orientation does not disrupt bundlemer alignment and hydrophobic-core packing, allowing formation of persistent rods with micrometre-scale contour lengths. Furthermore, the data suggest distinct mechanistic features: whereas BNDL29 polymerizes through conventional step-growth chemistry, BNDL15-TR rods grow predominantly by supramolecular folding and rod extension, achieving aspect ratios unattainable by purely chemical polymerization routes. These studies also open the possibility that other coiled coils could be truncated in a similar manner to impart sticky-end mediated polymerization, expanding the design space for programmable peptide assemblies. Together, these findings underscore the versatility of these bundlemer designs and provide a blueprint for creating minimalist yet robust peptide nanorods with potential applications in electronic, filtration, and responsive biomaterial applications.

## Conflicts of interest

There are no conflicts to declare.

## Supplementary Material

NR-017-D5NR03269E-s001

## Data Availability

The data supporting this article have been included as part of the supplementary information (SI). Supplementary information: detailed synthesis protocols, materials characterization data, and additional cryo-TEM images. See DOI: https://doi.org/10.1039/d5nr03269e. Additional data related to this study are available from the corresponding author upon reasonable request.
