# Supplementary material for: Supramolecular assembly of unstructured peptides into rigid bundlemer polymers
Source: Nanoscale. 2025 Oct 23;17(45):26057–61. doi: 10.1039/d5nr03269e (PMC12584133; doi:10.1039/d5nr03269e)
Supplement: NR-017-D5NR03269E-s001 [file NR-017-D5NR03269E-s001.pdf]

Supplementary Information (SI) for Nanoscale.
This journal is © The Royal Society of Chemistry 2025

# Supramolecular Assembly of Unstructured Peptides into Rigid Bundlemer Polymers

Joshua E. Meisenhelter,[a] Matthew Langenstein,[b] Jacquelyn E. Blum,[c] Dai-Bei Yang,[c] Darrin J. Pochan,[b] Jeffery G. Saven,*[c] and Christopher J. Kloxin*[a,b]

[a] Department of Chemical and Biomolecular Engineering, University of Delaware, 150 Academy Street, Newark, DE 19716, United States

[b] Department of Materials Science and Engineering, University of Delaware, 201 DuPont Hall, Newark DE 19716, United States

[c] Department of Chemistry, University of Pennsylvania, 231 S. 34th Street, Philadelphia, PA 19104, United States

## Table of Contents

Reagents and Materials ..... 2
Synthetic Methods ..... 2
On-resin addition of 4-maleimidobutyric acid to BNDL29 and BNLD15-TR: ..... 3
Synthesis of Polybundlemer Rods ..... 3
Characterization of Thiol–maleimide Peptide Conjugation ..... 9
Characterization of Peptide Materials ..... 4
Circular Dichroism (CD) Spectroscopy ..... 6
Cryogenic Transmission Electron Microscopy (Cryo-TEM) ..... 9

## Reagents and Materials

All chemicals were obtained from Fisher Scientific and used without further purification unless otherwise specified.

## Synthetic Methods

**Peptide synthesis**: All peptides listed in Table S1 were synthesized using standard Fmoc-solid phase peptide synthesis (SPPS) using previously described methods.[1] Synthesis was performed on a Liberty Blue microwave-assisted synthesizer (CEM) using Fmoc-protected amino acids, diisopropylcarbodiimide (TCI, 8× excess), and OxymaPure (CEM, 4× excess) in N,N-dimethylformamide (DMF). Couplings were carried out at 90 °C for 2 minutes and repeated twice. Fmoc deprotection was performed with 20% piperidine (Sigma-Aldrich) in DMF at 75 °C.

Peptides were cleaved from the resin using a trifluoroacetic acid (TFA, Honeywell) cleavage cocktail (92.5/2.5/5% TFA/water/triisopropylsilane with 50 mg/mL phenol and 50 mg/mL 1,4-dithiothreitol) for 3 hours. Crude peptides were precipitated with diethyl ether and purified by reverse-phase HPLC (Waters 2535) using a 5 to 95% acetonitrile gradient in water with 0.1% TFA. Final products were confirmed by UPLC-ESI-MS (Xevo, Waters) (see Figure S2), and purified peptides were lyophilized to yield white powders.

**Table S1** – Amino acid sequences of all peptides and their modifications, including the calculated molecular weight of the peptides.

| ID | Sequence | | | | | | Molecular Weight (g/mol) |
|---|---|---|---|---|---|---|---|
| BNDL29 | | DEEIRRM | AEEIRQM | AERIQQM | AEQIQQE | A | 3559.97 |
| BNDL29-C | C | DEEIRRM | AEEIRQM | AERIQQM | AEQIQQE | A | 3663.12 |
| BNDL29-Mal | Mal | DEEIRRM | AEEIRQM | AERIQQM | AEQIQQE | A | 3726.13 |
| BNDL15-TR | | DEEIRRM | AEEIRQM | A | | | 1876.13 |
| BNDL15-TR-C | C | DEEIRRM | AEEIRQM | A | | | 1979.27 |
| BNDL15-TR-Mal | Mal | DEEIRRM | AEEIRQM | A | | | 2042.29 |

## On-resin addition of 4-maleimidobutyric acid to BNDL29 and BNLD15-TR:

A solution of 4-maleimidobutyric acid (91.6 mg, 5 equiv, Combi-Blocks), HCTU (206.9 mg, 5 equiv, Chem-Impex), and diisopropylethylamine (348.4 μL, 10 equiv, Fisher) was prepared in 4 mL of DMF and pre-activated for 10 minutes. The mixture was then added to resin-bound peptide (0.1 mmol, 1 equiv) and allowed to react at room temperature for 2 hours. After filtration, the resin was washed sequentially with dichloromethane (3×), methanol (2×), and dichloromethane (3×). The coupling and wash steps were repeated once with freshly prepared reagent. Mass [m/z]: Peptide Mass+166.16 g/mol (see UPLC-ESI-MS data).

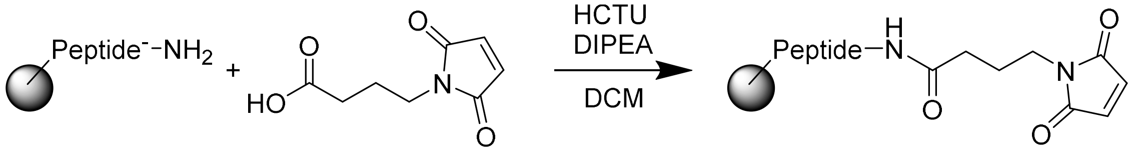


**Figure S1 – Schematic representation of on-resin N-terminal maleimide functionalization via coupling of 4-maleimidobutyric acid to a peptide.**

## Synthesis of Polybundlemer Rods

For this study, the thiol–maleimide coupling was carried out under conditions designed to decouple the covalent chemistry from the supramolecular assembly process. Lyophilized peptides were dissolved in deionized water (10 mM, unadjusted pH ≈ 5) and equimolar cysteine- and maleimide-functionalized variants were combined (final concentration 5 mM each). The reaction mixture was heated at 80 °C for 4–16 h to drive complete thiol–maleimide conversion in the absence of higher-order assembly. This elevated temperature ensured efficient cross-linking of the unstructured, two-heptad peptides before rod formation could occur, reducing the likelihood of trapping unreacted monomers within the polymer backbone. Following the covalent coupling step, the solution was cooled to room temperature and incubated for at least 24 h, allowing supramolecular folding and rod growth to proceed under mild conditions. This protocol contrasts with our earlier approach (Ref. 16), where the reaction was performed at room temperature using pre-assembled coiled-coil bundles. The current strategy was therefore deliberately chosen to separate chemical polymerization from physical assembly, enabling the discovery of a distinct, assembly-driven growth mechanism.

# Characterization of Peptide Materials

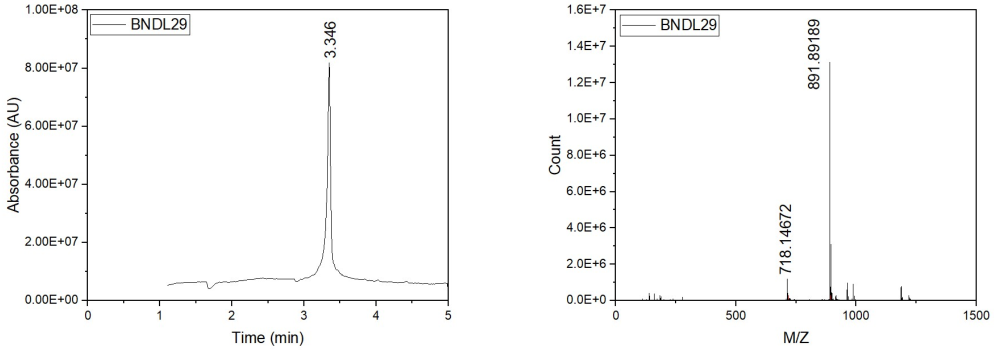


A

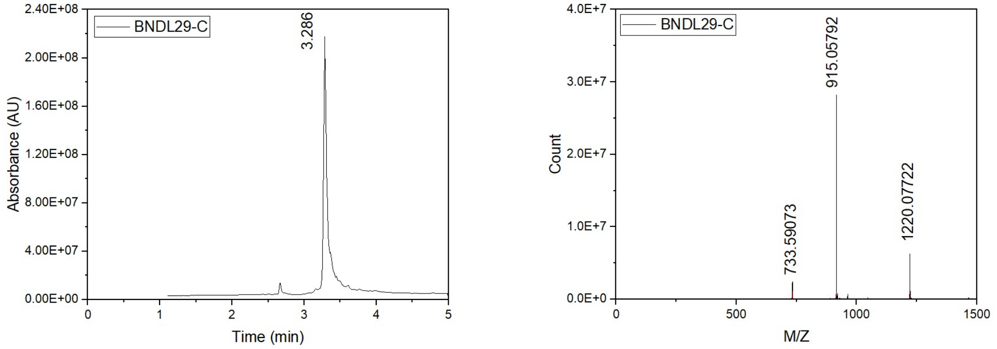


B

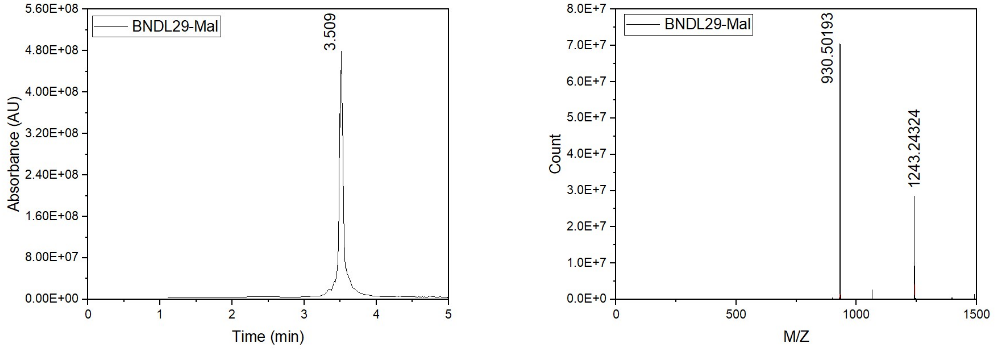


C

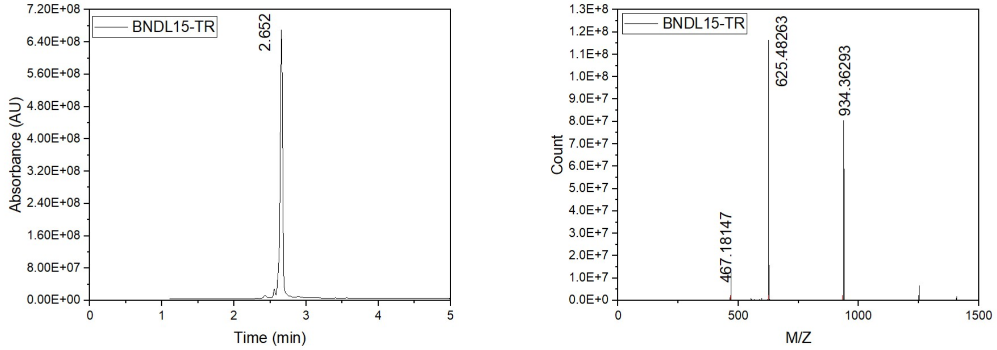


D

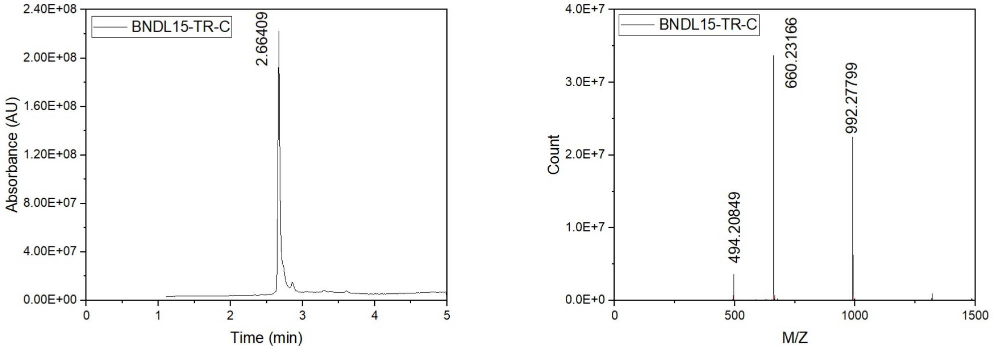


E

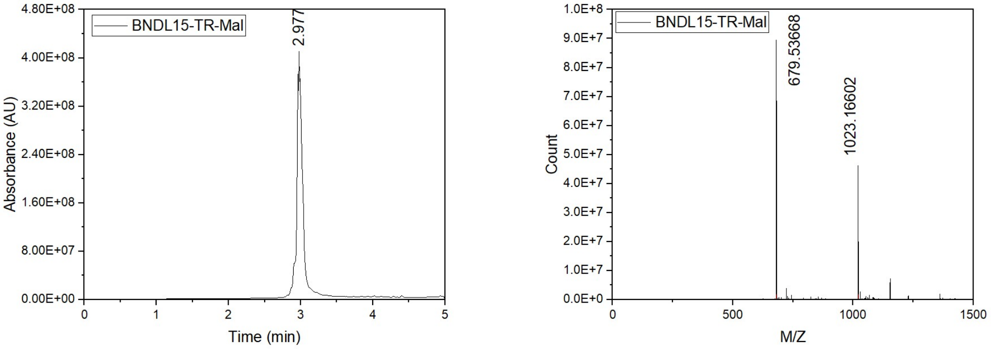


F

**Figure S2 – UPLC-ESI-MS Analysis of Peptides**. The left panels display ultra-high pressure liquid chromatography chromatograms of purified peptides. The right panels display the corresponding mass spectroscopy. A-F corresponds to BNDL29, BNDL29-C, BNDL29-Mal, BNDL15-TR, BNDL15-TR-C, BNDL15-TR-Mal, respectively.

## Circular Dichroism (CD) Spectroscopy

Peptide secondary structure was assessed using circular dichroism (CD) spectroscopy (Jasco J-1500), as previously described.[1] Lyophilized peptides were weighed gravimetrically, dissolved in deionized water to the desired concentration, and verified by UV-Vis spectroscopy at 280 nm (Thermo Scientific Nanodrop 2000C). Samples were heated from 10 °C to 90 °C at a rate of 1 °C/min, with CD spectra collected every 10 °C. Measurements were acquired in a 1 mm pathlength quartz cuvette over a wavelength range of 185–250 nm, using a 4 s digital integration time, 50 nm/min scanning speed, and averaging of three accumulations. Data were reported as concentration-corrected mean residue ellipticity (MRE), calculated as $MRE = CD / (l \cdot c \cdot n)$, where $CD$ is ellipticity (mdeg), $l$ is path length (cm), $c$ is peptide concentration (mol/L), and $n$ is the number of residues.[2]

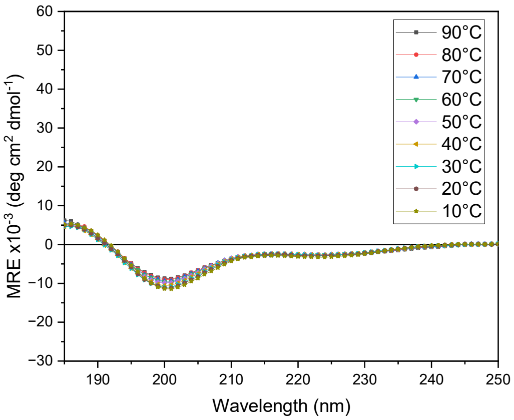

MRE x10$^{-3}$ (deg cm$^2$ dmol$^{-1}$)
Wavelength (nm)
90°C
80°C
70°C
60°C
50°C
40°C
30°C
20°C
10°C


A

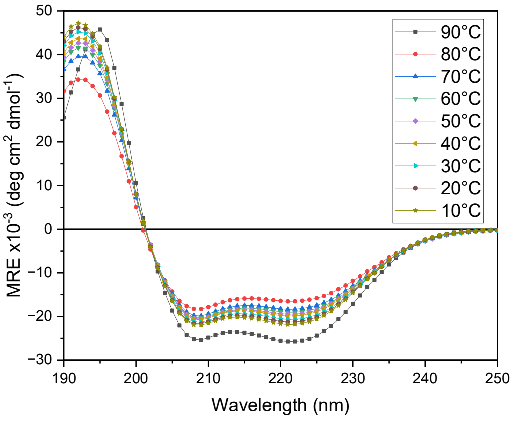

MRE x10$^{-3}$ (deg cm$^2$ dmol$^{-1}$)
Wavelength (nm)
90°C
80°C
70°C
60°C
50°C
40°C
30°C
20°C
10°C


B

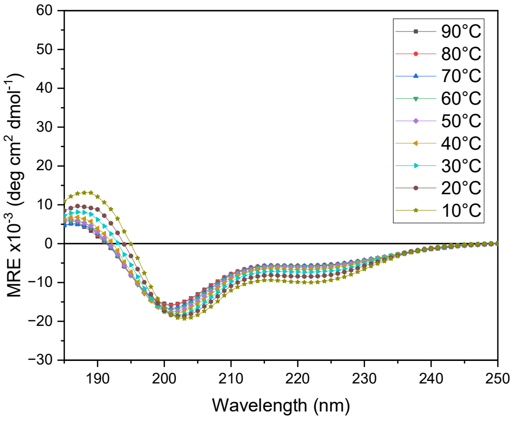

MRE x10$^{-3}$ (deg cm$^2$ dmol$^{-1}$)
Wavelength (nm)
90°C
80°C
70°C
60°C
50°C
40°C
30°C
20°C
10°C


C

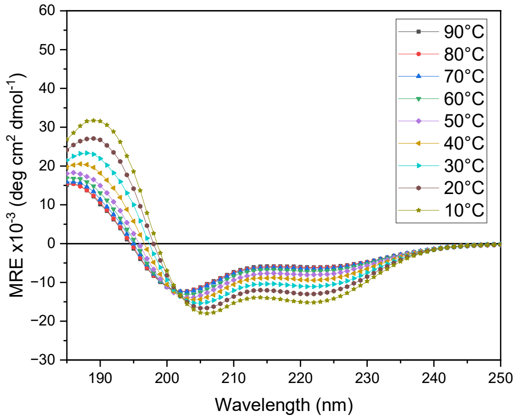

MRE x10$^{-3}$ (deg cm$^2$ dmol$^{-1}$)
Wavelength (nm)
90°C
80°C
70°C
60°C
50°C
40°C
30°C
20°C
10°C


D

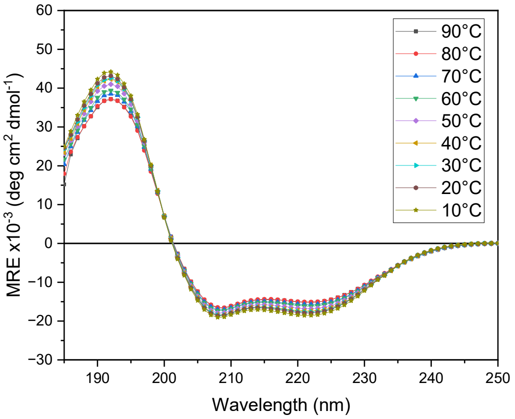

MRE x10$^{-3}$ (deg cm$^2$ dmol$^{-1}$)
Wavelength (nm)
90°C
80°C
70°C
60°C
50°C
40°C
30°C
20°C
10°C


E

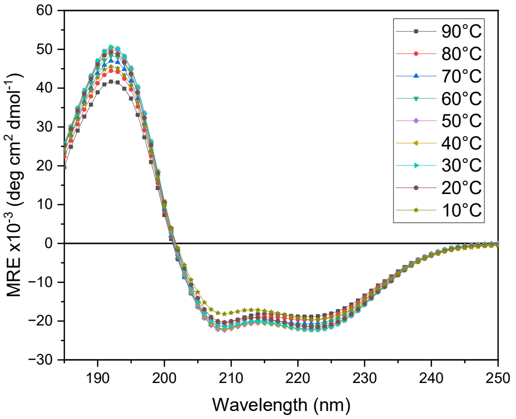

MRE x10$^{-3}$ (deg cm$^2$ dmol$^{-1}$)
Wavelength (nm)
90°C
80°C
70°C
60°C
50°C
40°C
30°C
20°C
10°C


F

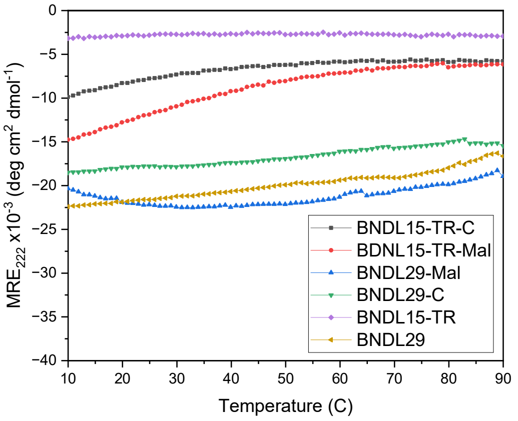


G

**Figure S3 - Circular dichroism analysis of BNDL15-TR and functionalized derivatives.** Peptides were prepared at 0.1 mM in deionized water without pH adjustment. Samples were cooled from 90 °C to 10 °C at a rate of 1 °C/min, with spectra collected every 10 °C for (A) BNDL15-TR, (B) BNDL29, (C) BNDL15-TR-C, (D) BNDL15-TR-Mal, (E) BNDL29-C, and (F) BNDL29-Mal. (G) Ellipticity at 222 nm was monitored continuously during the thermal ramp and plotted as a function of temperature for each peptide.

**Table S2 – Percent Helicity of Peptides.** Helical content was estimated from CD spectra collected at 20 °C using the BeStSel analysis server.[3,4]

| Peptide ID | Percent Helicity |
|---|---|
| **BNDL15-TR** | 1.0 |
| **BNDL15-TR-C** | 22.1 |
| **BNDL15-TR-Mal** | 15.8 |
| **BNDL29** | 100.0 |
| **BNDL29-C** | 100.0 |
| **BNDL29-Mal** | 100.0 |

## Characterization of Thiol–maleimide Peptide Conjugation

Stock solutions of BNDL15-TR-C and BNDL15-TR-Mal were each prepared at 10 mM in deionized water. Equal volumes were mixed to obtain a reaction solution containing 5 mM of each peptide. The mixture was incubated with shaking at 60 °C for 16 h. Mass spectrometry confirmed product formation, with peaks at 2.50 min (BNDL15-TR-C), 2.75 min (conjugated BNDL15-TR dimer), and 2.95 min (BNDL15-TR-Mal).

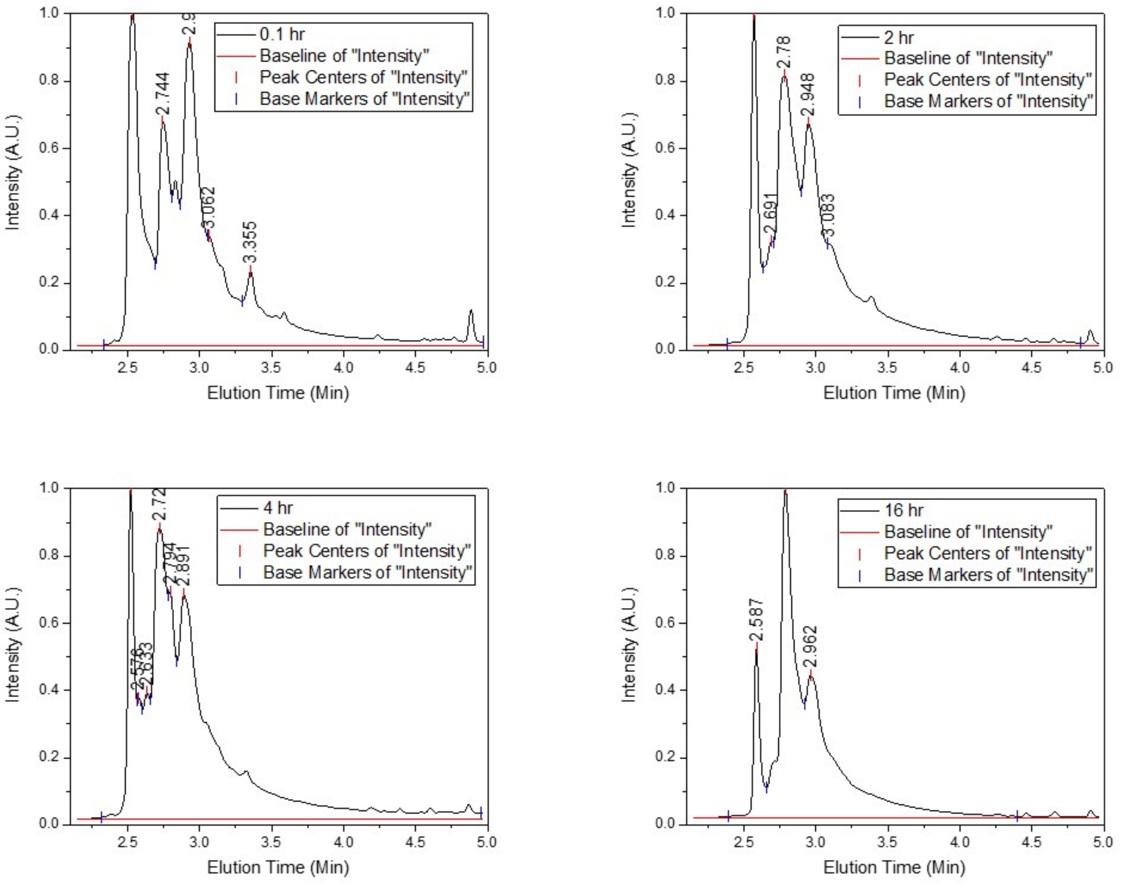


**Figure S4 – UPLC traces for mixture of BNDL15-TR-C and BNDL15-TR-Mal at 60°C taken at 0.1 (top left), 2 (top right), 4 (bottom left), and 16 (bottom right) hours of reaction time.** Peptides were prepared at 10 mM in deionized water without pH adjustment.

Conversion was estimated by integrating the area under peak 1 (BNDL15-TR-C) and normalizing to half of the total peak area, which corresponds to the initial concentration of BNDL15-TR-C. Conversion was defined as $\rho = 1 - [\text{BNDL15-TR-C}]/[\text{BNDL15-TR-C}]_0$. Peak 1 was chosen for analysis because it exhibited the least overlap with neighboring peaks; however, the calculation remains approximate due to residual convolution. Using this method, ~60% conversion was observed within 0.1 h at 60 °C, increasing to ~70% at 2–4 h and reaching ~80% after 16 h.

In addition to the cryo-TEM evidence of peptide assembly, we observed a pronounced increase in scattering intensity immediately after mixing BNDL15-TR-C and BNDL15-TR-Mal. Stock solutions of each peptide (10 mM in filtered deionized water) were prepared, and scattering intensity was

monitored at a 90° angle using a Wyatt NanoStar instrument.  The solution of BNDL15-TR-Mal alone showed no appreciable change over a 10-minute timescale.  By contrast, the BNDL15-TR-C/BNDL15-TR-Mal mixture displayed an immediate rise in scattering intensity that continued to increase over the course of the experiment (>6000 s). Occasional spikes in the count rate were attributed to dust or other insoluble particles.

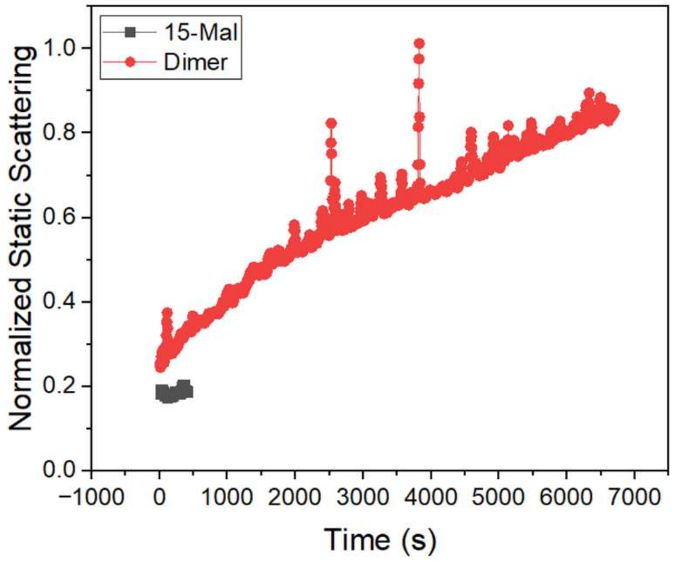


**Figure S5 – Normalized static light scattering of BNDL15-TR-Mal alone (15-Mal) and the BNDL15-TR-C/BNDL15-TR-Mal mixture (Dimer).** Stock solutions of each peptide (10 mM in filtered deionized water) were prepared and the scattering intensity was monitored at 90°.

## Cryogenic Transmission Electron Microscopy (Cryo-TEM)

Imaging was performed on a FEI Talos F200C (Thermo Fisher Scientific, Waltham, MA) using 200-mesh lacey carbon grids (Ted Pella, Redding, CA). Grids were plasma cleaned using a PDC-32G benchtop plasma cleaner (Harrick Plasma, Ithaca, NY) and used immediately. All grids were prepared using an FEI VitroBot (Thermo Fisher Scientific, Waltham, MA, USA). 4uL of the sample suspension was pipetted onto the grid inside a humidity chamber kept at 100% humidity to slow evaporation. The sample was allowed to diffuse into the lacey carbon for 30-60 seconds before being blotted with grade 595 filter paper (Ted Pella, Redding CA, USA). The grid was immediately plunged into liquid ethane to vitrify the sample and then swiftly transferred to liquid nitrogen. Grids were stored in liquid nitrogen for less than 2 hours before use.

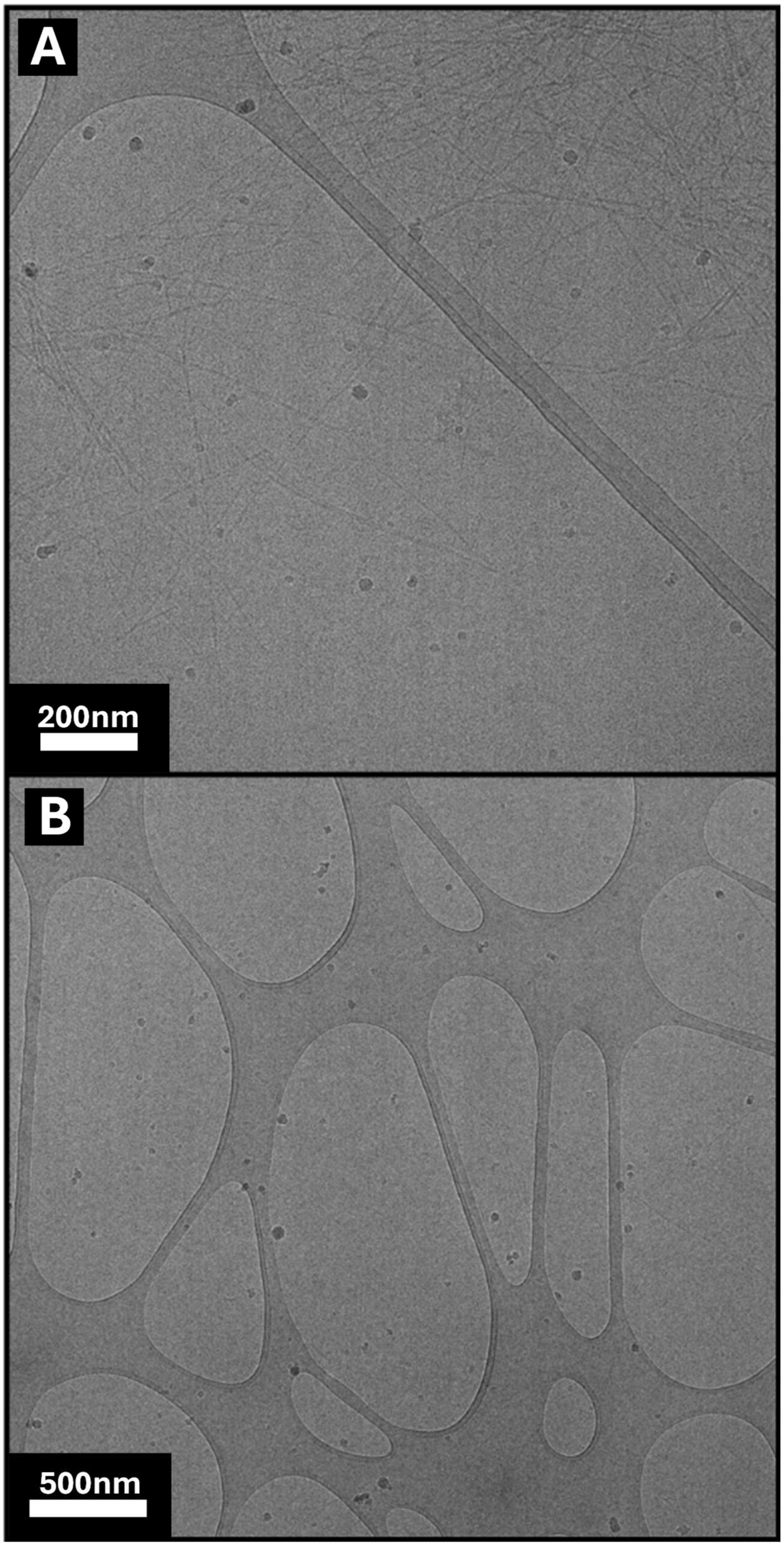


**Figure S6 – Additional cryo-TEM images of BNDL29-derived rods formed from N-terminally functionalized peptides with thiol and maleimide.** (A) High-magnification image showing uniform diameters of ~2 nm, consistent with a single coiled-coil bundlemer. (B) Low-magnification image revealing rod-like polymers extending 1–2 μm in length.

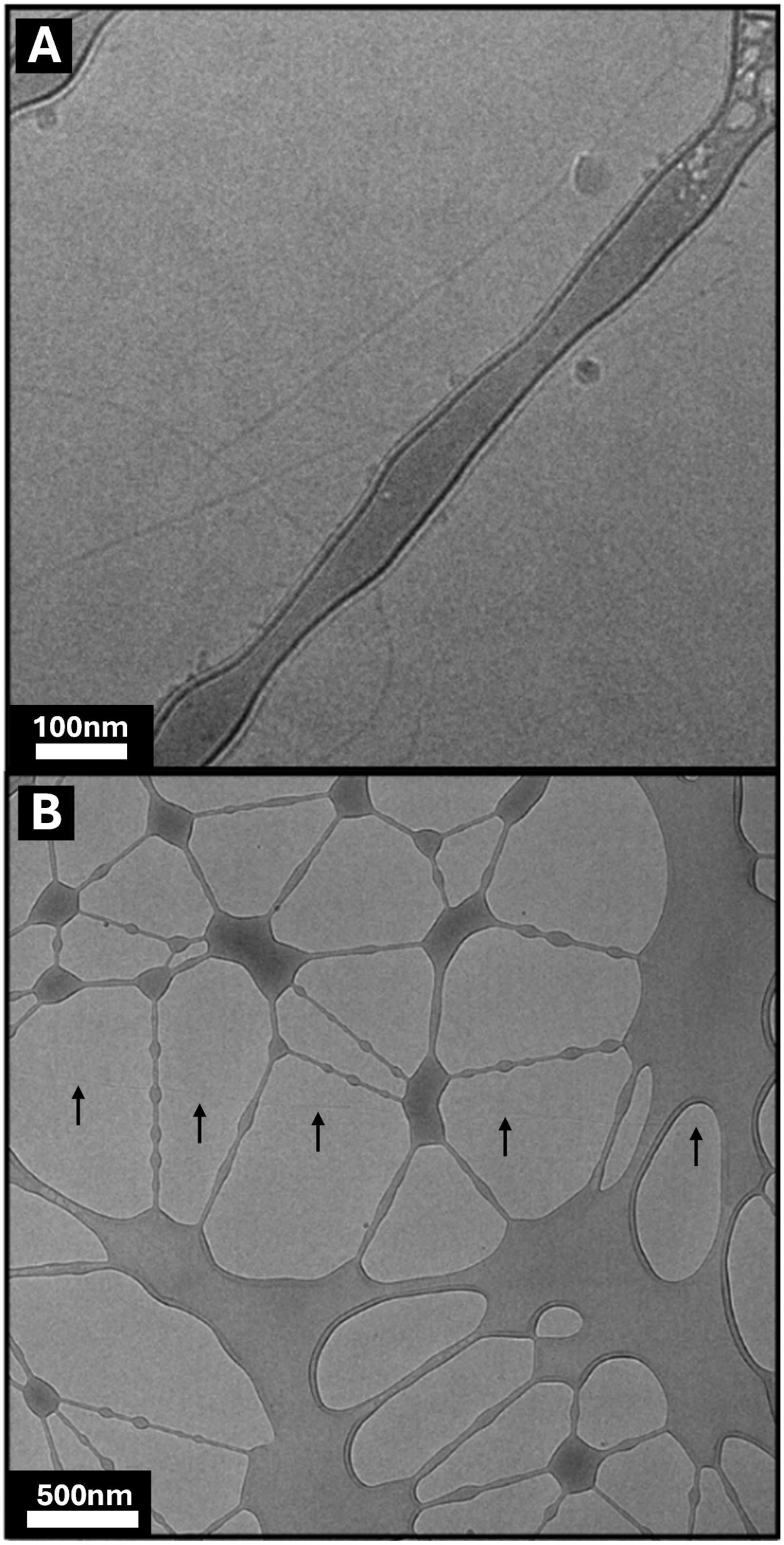


**Figure S7 – Additional cryo-TEM images of BNDL15-TR-derived rods formed from N-terminally functionalized peptides with thiol and maleimide.** (A) High-magnification image showing uniform diameters of ~2 nm, consistent with a single coiled-coil bundlemer. (B) Low-magnification image capturing a single polymer chain spanning the full field of view.
